# Factors predicting meat and meat products consumption among middle-aged and elderly people: evidence from a consumer survey in Switzerland

**DOI:** 10.1080/16546628.2017.1308111

**Published:** 2017-04-24

**Authors:** Alexandra Schmid, Doreen Gille, Patrizia Piccinali, Ueli Bütikofer, Magali Chollet, Themistoklis Altintzoglou, Pirjo Honkanen, Barbara Walther, Helena Stoffers

**Affiliations:** ^a^ Agroscope, Bern, Switzerland; ^b^ Nofima – Norwegian Institute for Food, Fisheries and Aquaculture Research, Tromsø, Norway

**Keywords:** Consumer survey, animal products, consumption frequency, consumer attitudes, aging, Switzerland

## Abstract

**Background**: An adequate diet contributes to health and wellbeing in older age. This is nowadays more important than ever since in industrialised countries the elderly population is growing continually. However, information regarding the consumption behaviour of older persons in Switzerland is limited.

**Objective**: The objective of this investigation was to explore how middle-aged and elderly Swiss view animal products in relation to diet and health, and what factors predict consumption frequency.

**Design**: A representative consumer survey among 632 people over the age of 50 years, living in the German-, French- and Italian-speaking regions of Switzerland was conducted.

**Results**: This paper presents the results related to meat and meat products consumption. Most participants consumed meat and meat products regularly. The majority of participants with low meat intake indicated that eating small amounts would be enough. Respondents judged fresh meat (except pork) to be healthier than meat products, and poultry to be the healthiest meat. Overall meat consumption frequency was predicted by language region, gender, household size, and BMI. Furthermore, participants’ opinion about healthiness, taste and safety of meat but not their adherence to the Swiss food pyramid was found to be correlated to the consumption frequency of individual types of meat.

**Conclusion**: Several factors have an impact on consumption frequency of meat and meat products in the middle-aged and elderly Swiss population and the importance varies according to the individual types of meat and meat products. The results show that the traditional food pyramid is not one of these factors for which reason new tools must be explored to support elderly people in regard to a healthy dietary behaviour.

## Introduction

A well-balanced diet is an important element for health and wellbeing through the whole life span. The same nutritional recommendations apply, in principle, to the healthy elderly and other adults. However, various physiological alterations that occur with aging (e.g. in body composition, gastrointestinal tract, water balance and bone health) affect the nutritional needs of elderly persons [1–3]. While energy requirements decrease with age, the necessary amount of micronutrients remains the same or even increases, so there is need for more nutrient dense foodstuffs and a careful selection of foods [4,5]. An adequate supply of high-quality protein is important to maintain muscle and bone health, to improve strength and physical function in elderly persons, and to help prevent sarcopenia [6–9]. Fresh meat corresponds well to the nutritional requirements of the older population as it contains a wide range of important nutrients, such as essential fatty acids, vitamins and minerals, as well as high biological value proteins [10–13]. However, various epidemiological studies link red meat and meat products with colorectal cancer [14–16], for which reason a limited intake is recommended [17]. Additionally, meat is a source of saturated fatty acids and cholesterol [18], both of which were believed over the last few decades to increase the risk of heart disease. Although the recommendation about dietary cholesterol has become obsolete and the role of saturated fatty acids is currently being reconsidered [19–21], few consumers are up to date regarding these topics.

In Switzerland, dietary recommendations are given by means of the Swiss food pyramid [22]. The recommendation regarding meat is one portion per day, alternating with other protein sources such as eggs, tofu, fish and cheese. A portion of meat is defined as 100–120 g. For elderly people, special emphasis is placed on ensuring an adequate protein supply, which is considered to be most efficiently accomplished by consuming foods of animal origin [22].

The age distribution has changed substantially in Switzerland in the 20th century. From 1900 to 2011, the proportion of people older than 64 years increased from 5.8% to 17.2%, and the proportion of people aged 80 years and older from 0.5% to 4.8%. Increased life expectancy and decreased birth rates are the main causes of these changes, and the trend is expected to continue in the 21st century. By 2060, the percentage of persons older than 64 years has been projected to reach 28% in Switzerland [23]. However, increased life expectancy is not accompanied with decreased disease prevalence but with a longer period of morbidity [24].

In light of the impact of nutrition and the projected growth of the elderly population in Switzerland and other industrialised countries, it is important to understand the dietary habits, attitudes towards various foodstuffs and nutritional knowledge of this population group. Unfortunately, there have been few studies dealing with these issues in Switzerland. Focusing on animal products, we were interested in the consumption behaviour of the elderly and how they view animal products in relation to their diet and health. We conducted a consumer survey among people aged 50 years and older living in Switzerland. The aim of the survey was to identify consumption frequencies and beliefs, and knowledge related to various animal products. In this paper, we focus on meat and meat products. In light of a generally rather high meat consumption in Switzerland (52.4 kg per person per year [25]), we also look at the motives underlying consumption avoidance or low consumption of meat and meat products in middle-aged and elderly people. Additionally, we investigate which factors predict consumption frequency of meat and meat products in this population group.

## Material and methods

### Participants and data collection

Quantitative data were collected through a questionnaire-based consumer survey in a representative sample in Switzerland. The sampling was performed by LINK institute for market and social research (Lucerne, Switzerland). A two-stage random-quota sampling procedure, with gender, age and region as main control variables, was applied. In stage one, telephone numbers were randomly chosen from the Swiss telephone directory. In the second stage, participants from the contacted households were established according to predetermined quotas. The age of the population was defined as 50 years or older, and the following regional allocation was targeted: German-speaking part of Switzerland, 50% of participants; French-speaking part of Switzerland, 30% of participants; Italian-speaking part of Switzerland, 20% of participants. The number of Italian-speaking participants was intentionally over-represented to assure enough statistical power. Participants had to live at home and not in an institution. A total of 726 persons were recruited by phone.

Data collection was performed between September and November 2012. The questionnaire was available online and in paper-and-pencil format in the three official languages of Switzerland (German, French, Italian). The link to the web or the paper version of the questionnaire, together with a stamped return envelope, were mailed to the participants, and they were asked to fill out the questionnaire within two weeks. After this period, a reminder was sent out to non-responders. Participants received a shopping voucher as an incentive.

We did not seek an ethic’s committee approval because, in Switzerland, it is not necessary for this type of study.

### Questionnaire

The questionnaire was originally developed in English and then translated into German, French and Italian. It was pre-tested with a small group of volunteers, who were explicitly asked to comment on the clarity of the questions. Thereafter, some items were re-formulated for enhanced precision and clarity. Finally, two native speakers checked the three language-versions of the questionnaire. Participants answered a total of 50 questions regarding health and nutrition [26], milk and milk product consumption [27,28], meat and meat product consumption and socio-demographic variables. The present paper focuses on factors predicting consumption frequency of meat and meat products, and on motives underlying consumption avoidance or low consumption.

Consumers were asked to report the frequency of their consumption of beef, veal, pork, poultry, horse and lamb meat as well as the meat products cooked sausages, raw sausages, cooked cured products and raw cured products using a 7-point frequency scale with the amounts ‘never’, ‘less than once a month’, ‘1–3 times per month’, ‘once a week’, ‘several times per week’, ‘once a day’ and ‘several times per day’. For the four meat product categories well known product examples were given in brackets. The following scale was used to transform the data into portions per week: never = 0, less than once a month = 0.125, 1–3 times per month = 0.5, once a week = 1.0, several times per week = 2.0, once a day = 7.0 and several times per day = 14.0. Missing answers were not taken into account. All transformations emanate from once a week = 1 portion. We made conservative choices for the transformation of portions per week into values and have chosen this approach to be conform to previous investigations [26,27].

To investigate which factors affect consumption frequency, and because meat intake is generally rather high in Switzerland, persons not eating meat or meat products or eating them only rarely (less than once a week) were asked to give reasons for their behaviour. The motives for low consumption were investigated with a 5-point Likert scale ranging from 1 = ‘totally disagree’ through 3 = ‘neither disagree nor agree’ to 5 = ‘totally agree’. The provided reasons were: ‘animal disease (e.g. avian flu)’, ‘small amounts are enough for me’, ‘afraid of microorganisms (e.g. virus, bacteria)’, ‘does not taste good’, ‘because of cholesterol’, ‘because of salt content’, ‘is too expensive’, ‘has too much fat’, ‘because of animal welfare (animal husbandry, animal transport)’, ‘because of residues in meat (e.g. antibiotics, hormones, dioxins)’, ‘out of religious believes’, ‘afraid to gain weight’, ‘ecological reasons (e.g. sustainability, long transportation routes)’, ‘on advice of another person (e.g. physician, dietician)’, ‘afraid of imitations’ and ‘visible blood’.

All participants were asked to evaluate beef, pork, and poultry meat in regard to five items (taste, fat content, safety, digestibility and preparation effort), each to be answered on a 5-point Likert scale (1 = ‘not at all’, 5 = ‘very’). Additionally, they had to give their opinion about the estimated healthiness of various types of meat and meat products on a 5-point Likert scale ranging from 1 = ‘not healthy at all’ through 3 = ‘neither healthy nor unhealthy’ to 5 = ‘very healthy’.

In the general part of the questionnaire, participants were asked several questions about nutrition and health. They had to indicate whether they were aware of and followed the official Swiss food pyramid by the Swiss Society for Nutrition (yes/no) [22]. Furthermore, they were prompted to specify on a 5-point Likert scale (1 = ‘not at all important’; 5 = ‘very important') how important healthy nutrition is for them and how healthy they rate their own nutrition (1 = 'not at all healthy'; 5 = ‘very healthy'). They also had to state (yes/no) whether they follow a specific diet (e.g. vegetarian, vegan, weight reduction, food intolerance).

At the end of the questionnaire, the following socio-demographic variables were assessed: gender, age, income, education, number of persons living in the household and level of employment (full time, part time, unemployed, retired, housewife/man). Weight and height were self-reported in the questionnaire and used to calculate body mass index (BMI).

### Data analysis

Statistical analysis was performed using Systat® version 13.0 (Systat Software Inc., Richmond, CA, USA). Descriptive analyses were applied for the characterisation of the dependent variables. To detect significant differences in consumption frequency between groups (e.g. gender, BMI, Swiss food pyramid adherence), the nonparametric Kruskal–Wallis test was used and pairwise comparisons were conducted with the Conover–Inman test. Significant differences between the answers for different types of meat and meat products (e.g. reasons for low consumption, estimations about healthiness) were identified using the Wilcoxon signed-rank test. The Bonferroni correction was applied to adjust for multiple testing. Both above tests were chosen because they are independent of normal data distribution. The Kruskal–Wallis test is appropriate for comparing two or more independent samples and the Wilcoxon signed-rank test is used for related samples [29,30]. Furthermore, relationships between numerous categorical and continuous independent variables and consumption frequency or health estimate as dependent variables were investigated using the General Linear Model (GLM) with analysis of covariance (ANCOVA) design. A stepwise backward elimination of non-contributing variables from the model was applied. In a further step, Fisher’s LSD test was used for pairwise comparison of individual categorical variables. This approach allows to include a large amount of possible predictors and to identify the relevant factors. GLM with ANCOVA design has the advantage that both continuous and categorical predicting variables can be included [31]. Differences were considered statistically significant at a level of *p *≤ 0.05.

## Results

### Socio-demographic profile

Overall, 646 of the 726 persons recruited by phone participated in the survey. After exclusion of four questionnaires because the respondents were <50 years and of ten questionnaires because of incompleteness, 632 completed questionnaires remained for analysis. Respondents were between 50 and 81 years old (mean 62.9 years) and most were of Swiss nationality (92%). Table 1 describes the sociodemographic distribution of participants and compares it with available data of the general population in this age group. Of the participants, 50.3% answered the German, 30.4% the French and 19.3% the Italian questionnaire. Respondents were representative of the Swiss population of 50 years and older for age, gender and language region. Italian-speaking participants were intentionally over-represented for statistical reasons.

**Table 1.** Characteristics of the 632 respondents and of the general population in the same age group in the year 2012 [32–34].

	n (%) of survey	% of general population [32–34]
Total sample size	632 (100)	
Sex		
Women	323 (51.1)	51.4
Men	309 (48.9)	48.6
Age		
50–60 years	282 (44.6)	46.0
61–70 years	209 (33.1)	32.7
71–80 years	141 (22.3)	21.3
Nationality		
Swiss	581 (91.9)	84.3
Other	42 (6.6)	15.7
Swiss and other nationality	8 (1.3)	na
No data	1 (0.2)	
Education		
Low (compulsory school and equivalent)	82 (13.0)	na^a^
Medium (professional education and equivalent)	280 (44.3)	na^a^
High (university and equivalent)	248 (39.4)	na^a^
Other	16 (2.5)	na^a^
No data	6 (0.9)	
Type of household		
1 person	163 (25.8)	24.6^b^
2 persons	321 (50.8)	44.6^b^
3 or more persons	145 (22.9)	31.8^b^
No data	3 (0.5)	
BMI (calculated)		
<18.5	13 (2.1)	2.5^c^
18.5≤BMI<25	310 (49.1)	44.8^c^
25≤BMI<30	226 (35.8)	38.2 ^c^
≥30	70 (11.1)	14.6^c^
No data	13 (2.1)	

na, Data not available.

^a^ Educational levels and age categories vary between current survey and national surveys.

^b^ National data for the age group ≥45 years in 2011.

^c^ National data for the age group ≥55 years.

### Frequency of meat and meat product consumption

**Figure 1.**
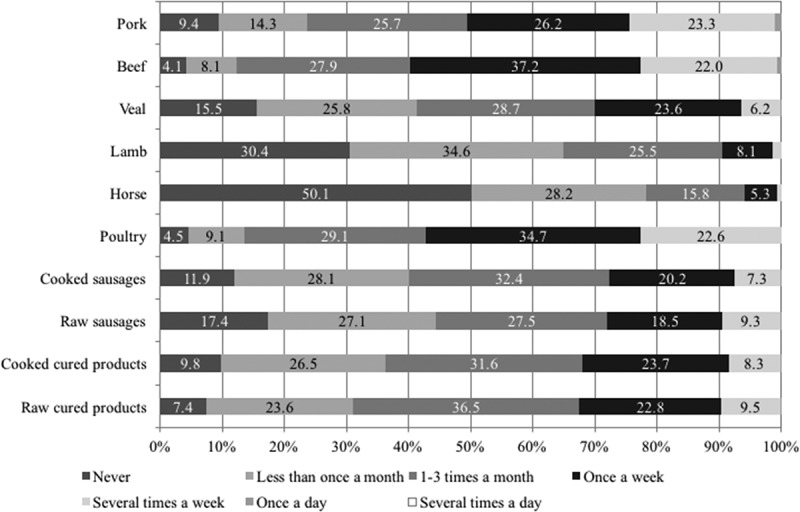
Percentages of consumption frequencies of meat and meat products of all respondents (*n* = 627). The option ‘several times a day’ was never chosen.

Most participants consumed meat and meat products regularly. Only 19 women and 15 men (34 participants; 5.4%) indicated they were ovo-lacto vegetarians or vegans. Figure 1 shows the consumption frequency of different kinds of meat and meat products as stated by the participants of the survey. Pork, beef and poultry were consumed most often: 50.6% (pork), 59.8% (beef) and 57.3% (poultry) of the participants indicated that they consumed these meats at least once a week. Not surprisingly, lamb and horsemeat were consumed least often, with 30.4% and 50.1% of the participants reporting that they never eat lamb or horse, respectively. The various meat products were all consumed about equally seldom: roughly 70% of the respondents eat the various types less than once a week.

Interestingly, only 12 of the 34 self-identified vegetarians declared that they never eat meat. The other 22 ‘vegetarians’ indicated to consume meat, most commonly poultry, from less than once a month to once a day. Men consumed meat and meat products significantly more often than women (overall mean of 7.2 [SD 3.6] vs. 5.3 [SD 2.7] portions/week, *p *< 0.001), and this was applicable for all types of meat except poultry. Persons adhering to the Swiss food pyramid (38% of the respondents) consumed meat significantly less often than persons not adhering to it (mean of 5.7 [SD 3.2] vs. 6.6 [SD 3.5] portions/week, *p *< 0.001). Especially, pork meat, cooked sausages and raw sausages are less frequently consumed by these persons. In contrast, persons who stated to know the Swiss food pyramid (71% of the respondents) did not have a significantly lower consumption frequency of meat compared to respondents not knowing the Swiss food pyramid (mean of 6.1 [SD 3.3] vs. 6.5 [SD 3.6] portions/week, *p *= 0.16). Persons with a BMI ≤25 kg/m^2^ (51% of respondents) consumed meat significantly less often than persons with a BMI >25 kg/m^2^ (5.7 [SD 3.4] vs. 6.7 [SD 3.3] portions/week, *p *< 0.001).

### Reasons for rarely eating meat and meat products

Participants not eating red meat (lamb, pork, beef, veal and horse), poultry or meat products (cooked sausages, raw sausages, cooked cured and raw cured meat products) – or consuming them only seldom (less than once a week) – were asked to give reasons for this behaviour. A total of 267 persons (42% of all participants) fulfilled this criteria for meat products and answered the relevant question. Of all participants, 212 (33%) answered the comparable question pertaining to white meat (poultry) and 176 (28%) answered the similar question regarding red meat.

Mean values (and SD) of participants’ agreement and disagreement with reasons for low consumption of meat products, red meat and white meat (poultry) expressed on the 5-point Likert scale are given in Table 2. The main findings were similar for red meat, meat products and poultry: high agreement was expressed for ‘small amounts being enough’ as well as ‘because of residues’ as reasons for low consumption and high disagreement was expressed for ‘religious reasons’. The mean level of agreement did not differ significantly between red meat, white meat and meat products for animal welfare reasons, ecological reasons and religious reasons. However, when asked whether cholesterol content, fat content and price were reasons to eat small amounts, significant variations in the answers between all three product categories emerged. Significantly more respondents agreed to worry about high cholesterol content in meat products compared to red meat or poultry and the same pattern was seen in regard to fat content. The price was a more important reason for rarely eating red meat than it was for meat products or poultry. Consuming only small amounts of poultry was less the result of advice from other people than it was the case in red meat and in meat products. Finally, the reason ‘afraid to put on weight’ received significantly more agreement by respondents in regard to meat products than in regard to poultry.

**Table 2.** Participants’ agreement (mean and SD) with reasons for low consumption (< once a week) of meat products, red meat and white meat (poultry) given on a 5-point Likert scale (1 = ‘totally disagree’, 2 = ‘rather disagree’, 3 = ‘neither disagree nor agree’, 4 = ‘rather agree’, 5 = ‘totally agree’).

	Meat products (*n* = 267)		Red meat (*n* = 176)		White meat (poultry) (*n* = 212)	
	Mean	SD	Mean	SD	Mean	SD
Animal diseases	2.34	1.28	2.36	1.25	2.64	1.29
Small amounts are enough	3.85	1.17	3.91	1.23	3.88	1.13
Afraid of microorganisms	2.44	1.28	2.48	1.30	2.75	1.33
Taste not good	2.59	1.26	2.41	1.27	2.35	1.24
Because of cholesterol	3.30^a^	1.25	2.92^a^	1.39	2.30^b^	1.21
Because of salt content	3.44	1.20	NA	NA	NA	NA
Too expensive	2.72^a^	1.16	3.16^b^	1.33	2.29^c^	1.15
Too much fat	3.73^a^	1.13	2.92^b^	1.27	2.06^c^	1.08
Because of animal welfare	2.95	1.35	3.09	1.38	3.00	1.44
Because of residues	3.32	1.34	3.35	1.37	3.21	1.37
Religious believes	1.55	0.96	1.61	1.37	1.62	1.00
Afraid to gain weight	2.72^a^	1.32	2.33^ab^	1.24	2.06^b^	1.20
Ecological reasons	2.89	1.35	2.99	1.45	2.96	1.42
Afraid of imitation	2.89	1.31	NA	NA	NA	NA
On advice of another person	2.40^a^	1.27	2.18^ab^	1.21	1.97^b^	1.11
Visible blood	NA	NA	2.23	1.37	2.01	1.15

NA, not asked.

^a,b,c^ Mean values within a row with unlike superscript letters were significantly different (*p *< 0.05) according to the Wilcoxon signed-rank test with Bonferroni correction for multiple testing.

### Evaluation of beef, poultry and pork

Participants were asked to evaluate beef, poultry, and pork meat with regard to the attributes taste, fat content, safety, digestibility, and preparation effort. The three types of meat were rated differently in all attributes except for preparation effort, which was categorized similarly for beef and pork (Table 3). Respondents were of the opinion that beef has the best taste and is the safest of the three types of meat. The taste of pork meat was the least appreciated and pork was indicated to be fattier and less digestible than the other two types of meat. Poultry was judged to be leaner, easier to prepare, better digestible but less safe compared to the other two meats.

**Table 3.** Participants’ estimation (mean and SD) on beef, pork, and poultry meat in regard to the five items taste, fat content, safety, digestibility, and preparation effort given on a 5-point Likert scale ('not at all' = 1, 'rather not' = 2, 'neither/nor' = 3, 'rather' = 4, 'very' = 5) (*n* = 593).

	Beef		Pork		Poultry	
	Mean	SD	Mean	SD	Mean	SD
‘tastes … good’	4.20^a^	0.75	3.85^c^	0.88	4.07^b^	0.74
‘is … fatty’	2.44^b^	0.85	3.84^a^	0.80	2.20^c^	0.88
‘is … safe’	3.68^a^	0.82	3.41^b^	0.85	3.05^c^	0.95
‘… easy to digest’	3.52^b^	0.84	3.17^c^	0.92	4.04^a^	0.68
‘… easy to prepare’	3.45^b^	1.06	3.49^b^	1.02	3.65^a^	1.06

^a,b,c^ Mean values within a row with unlike superscript letters were significantly different (*p *< 0.003) according to the Wilcoxon signed-rank test with Bonferroni correction for multiple testing.

### Healthiness of different types of meat and meat products

**Figure 2.**
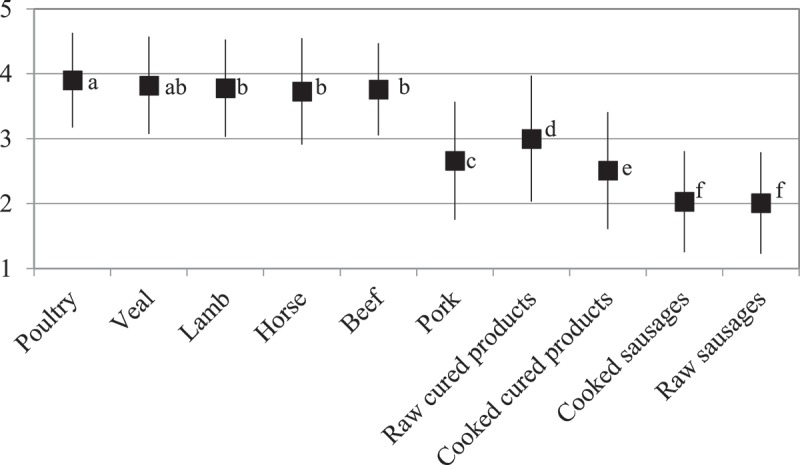
Respondents’ estimation of the ‘healthiness’ of various meat and meat products (*n* = 588). 1 = ‘not healthy at all’, 3 = ‘neither healthy nor unhealthy’, 5 = ‘very healthy’ (mean with standard deviation). Different superscript letters indicate significant differences (*p *< 0.05) according to the Wilcoxon signed-rank test with Bonferroni correction for multiple testing.

A total of 588 participants answered the question about the healthiness of different types of meat and meat products. Fresh meat was rated to be very or rather healthy by the majority of respondents (beef 74%, veal 74%, lamb 72%, horse 62% and poultry 79% of respondents) with the exception of pork, which 52% of the respondents judged to be not at all or rather not healthy. Figure 2 displays the respondents’ mean health estimations. The mean health estimation was significantly lower for pork (*p *< 0.001) and significantly higher for poultry (*p *≤ 0.03) compared to the other types of meat. Respondents also indicated that most meat products are not at all or rather not healthy (cooked sausages 80%, raw sausages 80% and cooked cured products 56% of respondents). For raw cured products respondents expressed diverging opinions: 35% believed these products to be not at all or rather not healthy compared to 37% rating them to be very or rather healthy. Overall, respondents judged fresh meat (except pork) to be healthier than meat products and poultry to be the healthiest meat.

Participants’ opinion about taste, fat content, safety, digestibility, and preparation effort of pork, beef, and poultry (see section above) explains the differing healthiness ratings of these meats only partly. In regard to pork meat, all five factors contribute to the healthiness estimation (multiple R^2^ = 0.234) but only taste (*p *= 0.028), fat content (*p *< 0.001) and digestibility (*p *< 0.001) did so significantly. The healthiness of beef meat is significantly predicted by participants’ opinion about taste (*p *< 0.001), safety (*p *= 0.017) and digestibility (*p *< 0.001) and not significantly by fat content of beef meat (multiple R^2^ = 0.191). The same four factors contribute to the participants’ estimation of the healthiness of poultry meat (multiple R^2^ = 0.228): participants’ opinion about taste (*p *< 0.001), fat content (*p *< 0.001), safety (*p *= 0.005), and digestibility (*p *= 0.002). With the exception of fat content all factors are positively correlated to the healthiness rating.

### Factors influencing meat consumption

The factors language region, gender, household size, education level, knowledge of and adherence to the Swiss food pyramid, opinion about the importance of a healthy nutrition and the estimation about the health of the participant’s diet as well as the co-variables age and BMI were included into the general linear model (GLM). Overall meat consumption was the dependent variable. GLM (with stepwise backwards elimination option) identified language region (*p *= 0.002), gender (*p *< 0.001), household size (*p *< 0.001) and BMI (*p *= 0.001) to be significant predictors for overall meat consumption (multiple R^2^ = 0.134) but rejected the co-variable age and the factors education level, the knowledge of and the adherence to the Swiss food pyramid as well as the opinion about the importance of a healthy nutrition and the estimation about the health of the participant’s diet. The estimated effects are presented in the online supplemental material (Table S1). An analysis of the individual parameters showed a significantly lower meat and meat products consumption frequency by Italian-speaking participants compared to German- and French-speaking participants. Furthermore, women ate meat and meat products less often than men.

According to McCarthy et al. [35,36] people’s attitude significantly influences consumption behaviour in regard to meat and the most significant determinants for pork, poultry and beef meat consumption are the attributes health, safety and taste. Therefore, we investigated whether the participants’ opinion about flavour, fat content, safety, digestibility, preparation effort, and health of beef, pork and poultry is related to the consumption frequency of these types of meat. We again included the socio-demographic parameters as well as the health related questions mentioned above into the model. Consumption frequency of beef is significantly predicted by participants’ opinion about taste (*p *= 0.004) and healthiness (*p *= 0.005) of beef but also by language region (*p *= 0.006), gender (*p *= 0.015), BMI (*p *= 0.023), and household size (*p *< 0.001), and non-significantly by their opinion about the safety of beef (multiple R^2^ = 0.158). The factors fat content, digestibility, preparation effort as well as age, education and whether participants adhere to the Swiss food pyramid and how important they rate a healthy nutrition were stepwise excluded by the model, indicating that they do not predict consumption frequency. The same factors were found to predict the frequency of pork consumption but additionally the factor ‘adherence to the Swiss food pyramid’ (*p *= 0.023) was included (multiple R^2^ = 0.238). A slightly different composition of predicting factors was identified for the frequency of poultry consumption (multiple R^2^ = 0.174). The factors comprise language region (*p *= 0.001), BMI (*p *< 0.001) and age (*p *= 0.026) as well as participants’ opinion about taste (*p *< 0.001) and safety (*p *= 0.018) of poultry. Additionally, fat content (*p *= 0.215) and preparation effort (*p *= 0.085) are part of the model. The estimated effects of the factors and co-variables can be found in the online supplemental material (Table S2–S4).

## Discussion

The present investigation aimed at assessing the predictors of consumption frequency of meat and meat products in participants from Switzerland aged 50 years and older and at identifying their attitudes in regard to meat and meat products. The results give valuable information pertaining to factors that predict the meat consumption of middle-aged and elderly people.

### Consumption frequency

In our study, the consumption frequency of beef, pork and poultry is the highest, with ≥50% of the participants consuming these types of meats at least once a week. This is in accordance with the 6th Swiss Nutrition Report and calculations of the Swiss meat industry trade organisation, although discrepancies in the consumption ranking of these three meat types exist [25,37]. This can be explained by the methodology used. The results of the present study do not represent an exact amount but rather a frequency of consumption estimated by the respondents. In contrast, the consumption data of the Swiss Nutrition Report and the data of the meat industry base on agricultural statistics of the amount of meat available. Other Swiss food frequency data are rare (e.g. [38,39]) and difficult to compare because meat categories as well as frequency categories are not always congruent. Since available data are similar to our results, we assume that the assessed consumption frequencies reflect reality in Switzerland as far as possible. However, since the frequency data were self-reported and not validated with other assessment methods, caution is advised in interpreting the results. Therefore, answers were predominantly used to separate low from high-consumers.

Of all participants, 5.4% claimed to be ovo-lacto vegetarians or vegans, which is in line with the 6% level in the whole population documented in a survey of the Swiss meat industry trade organisation [40]. However, in our survey less than half of the self-identified vegetarians actually abstained from meat consumption, resulting in only 1.9% strict vegetarians. Similar proportions were revealed in the Austrian Health Interview Survey 2006/2007 (2.2%) and the German National Nutrition Survey II (1.6%) in the whole population [41,42]. There seems to be a gap between declaration and behaviour of the respondents. Since the terms ‘vegetarian’ and ‘vegan’ were not defined in our survey because we presumed they were well known, misinterpretations might have led to unintentionally wrong answers. However, various studies have reported similar discrepancies, and there is evidence that self-identification results in higher estimates than behavioural reports [43–46]. There are several possible explanations for this phenomenon, such as imprecise definitions of a vegetarian diet, situational constraints, weak impulse control or social desirability [43].

### Factors influencing meat consumption frequency

GLM analysis identified the three socio-demographic factors language region, gender and household size as well as the co-variable BMI as predictors for consumption frequency. In our study, male respondents consumed all types of meat except poultry more frequently than their female counterparts. This is in agreement with the results of the European Prospective Investigation into Cancer and Nutrition (EPIC) study [47]. Even though consumption amounts varied distinctly between the centres in the 10 participating European countries, total meat intake in women was lower than in men in all cases. Lower meat consumption among women may be due to a greater health consciousness [48] but may also be linked to dislike and negative attitudes due to the bloodiness of meat and negative body feel after the consumption of meat [49]. In our survey, significantly more women than men indicated to adhere to the Swiss food pyramid, which is supporting the health consciousness hypothesis.

Our survey showed that participants adhering to the Swiss food pyramid have a significantly lower meat consumption frequency than participants not complying with the Swiss food pyramid. However, GLM did not identify ‘adherence to the Swiss food pyramid’ as a consumption predicting factor. An explanation for these findings may be the high percentage of women following the Swiss food pyramid.

Frequency of meat consumption was positively linked to household size in our study. In agreement with this, Fraser et al. [50] reported that single respondents eat meat less frequently than their married counterparts. Furthermore, Brunner and Casetti [51] recently found a positive albeit weak correlation of household size with meat and meat products consumption in a representative Swiss survey. In our study, Italian-speaking participants consumed meat and meat products less frequently than German- and French-speaking participants. Concordantly, the Swiss Health Surveys of 1992/93 and 2007 found a significantly lower percentage of frequent meat consumers (≥1 serving/d) in the Italian region of Switzerland compared to the German and French regions [39,46]. Finally, the results of our study are congruent with the findings of the EPIC study, which demonstrated that meat consumption is positively linked with increasing BMI [47].

Overall, these results suggest that the elderly population in Switzerland is comparable to the whole population – at least in regard to these four factors. However, the identified factors only explain about 13% of the variation in overall consumption frequency of meat and meat products. The examples of beef, pork, and poultry show that factors like taste, safety and healthiness also play an important role in regard to consumption frequency. Nevertheless, there have to be further factors influencing consumption frequency in the middle-aged and elderly Swiss population. Interestingly, the Swiss food pyramid, although being the predominant guide to a healthy diet in Switzerland, seems not to impact consumption frequency of meat and meat products. Our models explain less than 24% of consumption variance, which might not seem very much. However, results of this magnitude are common in nutrition studies [51–53] because nutrition is a complex issue with a multitude of factors influencing food choices and large differences between individuals.

### Reasons for low meat consumption

Participants with low meat consumption (less than once a week) were asked to indicate their reasons for this behaviour, separated for red meat, white meat and meat products. Substantial agreement was expressed for the reason ‘small amounts are enough for me’ irrespective of type of meat. On the one hand, this may reflect a reduction in food intake due to lower energy needs in the elderly population or a habitual low meat intake in this generation. On the other hand, it may describe the opinion of the respondents caused by the recommendations of the Swiss food pyramid [22] or by information acquired in print media or from the internet. Study results show that persons adhering to the Swiss food pyramid consumed meat significantly less often than persons not adhering to it. However, only 38% of respondents stated that they complied with the Swiss food pyramid, although more than 70% indicated that small amounts of meat were enough. Furthermore, multivariate GLM analysis did not identify ‘adhering to the Swiss food pyramid’ to be a factor predicting the frequency of meat consumption. This suggests that either the belief is based on other sources or the respondents’ statement indeed only reflects age-related reduction in food intake. Since results of the general part of the survey indicate that consumption of various food groups (e.g. vegetables, fruits, dairy products, cereals) are below the recommended level in more than 50% of the participants [26], we think that the respondents’ statement reflects a generally lower food intake in this age group.

Most participants with a low consumption of meat products stated the high fat and salt content, and a large proportion also indicated cholesterol content as a reason. The agreement for fat and cholesterol being a reason was significantly higher in meat products than in red meat or poultry. Salt as a reason was not assessed in these two (unprocessed) meat types. Meat products usually contain higher amounts of salt and/or fat than fresh meat, and both substances have been associated with negative health effects in the past [54,55]. This seems to be known among the participants of this survey. Congruently, participants rated the healthiness of meat products significantly lower than the healthiness of fresh meat.

Roughly half of the respondents agreed that they consumed meat and meat products not often because of ‘residues in meat’. Antibiotics, hormones and dioxins were mentioned as examples. This is probably related to articles and broadcasts picking up these topics periodically [56,57].

### Strengths and limitations

Some limitations of this study have to be addressed. People responding to such a survey may be more interested in health and nutrition than the general population. Additionally, self-reported food frequency intake data are liable to bias through under- and over-reporting [58]. However, the aim of the study was not to assess accurate nutritional intakes of the respondents. Rather, we wanted to study the relationships between meat intake and factors that influence the consumption of meat and meat products. Variations in response style such as extreme responding or use of the middle category on rating scales may lead to false variances and thus contribute to systematic error [59,60]. Furthermore, the accuracy of the answers may be influenced by ideas of socially desirable responses or general beliefs, which is a common phenomenon in consumer science [61]. The problem is multifaceted and difficult to get under control. To avoid as much of the problem as possible, we have put serious attention to the design of the questionnaire, based on previously published research and validated questionnaire items. Additionally, to reduce socially desirable responding, we clarified to the participants that there are no right or wrong answers and that the participation in the study is anonymous [59,60]. With regard to environmental sustainability consumption motives not only of animal products but also of alternative protein sources such as fish, tofu or pulses would be of interest. However, since we were predominantly interested in animal products (especially meat and dairy) and how the elderly view them in regard to their health and diet, we did not include questions regarding other protein sources in order to prevent a lengthy questionnaire.

This study also has its strengths. By concentrating on persons aged 50 years and older, the study provides in-depth information about a population group that is, although increasingly important, normally not the focus of attention, because most surveys target the whole population. The questionnaire items were compiled and optimised with the help of interviews and pre-tests to assure optimal comprehensibility and clarity of the questions. The study encompasses all three language regions in Switzerland, and rural as well as urban areas are covered. Other Swiss studies have often concentrated on only one or two language regions, without including the Italian-speaking part. Furthermore, providing the questionnaire in the three official languages of Switzerland as well as in a paper and online format is essential to collect reliable and representative responses, particularly with older participants that may be less comfortable with using on-line surveys.

## Conclusions

The results of this representative consumer survey show that although middle-aged and elderly people give similar reasons for low meat, poultry and meat products consumption, the importance of the different reasons varies between the three types of meat. Interestingly, the concern about residues such as hormones and antibiotics in meat is rather prominent and exceeds the concerns about fat and cholesterol in red meat and poultry. Farmers and manufacturers should therefore accept responsibility for transparency about their production methods in order to avoid losses in consumer confidence. Since the media might have increased the concern about residues, further research should clarify to what extend the media influences the meat consumption behaviour of elderly people and official bodies should evaluate how this can be used for their own purpose. Nonetheless, fat, cholesterol and salt contents are also important reasons to limit intake, especially in regard to meat products. Recommendations regarding cholesterol intake have changed substantially in the last few years and also the recommendations regarding fat intake are in flux. It is important, that official bodies promptly inform people when recommendations are adapted due to novel research results. However, as demonstrated by the cholesterol recommendations, it generally takes a long time until new information becomes accepted and consumers believe change. Although the Swiss food pyramid is the predominant guide for a healthy diet in Switzerland, it seems actually not to impact the consumption frequency of meat and meat products in the investigated population group. Thus, official authorities intending to regulate meat consumption should explore new tools to guide elderly people to a healthier behaviour.

## Supplementary Material

Supplemental Data
