# Supplementary material for: Factors predicting meat and meat products consumption among middle-aged and elderly people: evidence from a consumer survey in Switzerland
Source: Food Nutr Res. 2017 Apr 24;61(1):1308111. doi: 10.1080/16546628.2017.1308111 (PMC5404422; doi:10.1080/16546628.2017.1308111)
Supplement: Supplemental Data [file zfnr_a_1308111_sm6277.pdf]

Supplementary material

Table S1 Estimates of effects of predicting factors for overall consumption of meat and meat products (General Linear Model with ANCOVA)

Factor	B	Type III SS	df	Mean squares	F-Ratio	*p*-Value
Constant	3.214					
Language region		124.103	2	62.052	6.116	0.002
German	0.353					
French	0.411					
Italian	-0.764					
Gender		325.374	1	325.374	32.070	<0.001
Male	0.763					
Female	-0.763					
Household size		219.246	2	109.623	10.805	<0.001
1-person	-0.913					
2-person	0.121					
≥ 3-person	0.792					
BMI	0.114*	118.043	1	118.043	11.635	0.001

Multiple $R^2 = 0.134$

* Regression coefficient in the ANCOVA model for BMI

Table S2 Estimates of effects of predicting factors for pork consumption (General Linear Model with ANCOVA)

Factor	B	Type III SS	df	Mean squares	F-Ratio	*p*-Value
Constant	0.449					
Language region		10.702	2	5.351	7.137	0.001
German	0.180					
French	0.060					
Italian	-0.240					
Gender		5.037	1	5.037	6.718	0.010
Male	0.105					
Female	-0.105					
Household size		7.531	2	3.765	5.022	0.007
1-person	-0.185					
2-person	0.037					
≥ 3-person	0.148					
BMI	0.020*	3.067	1	3.067	4.091	0.044
Swiss food pyramid		3.911	1	3.911	5.217	0.023
Adhering to	-0.093					
Not adhering to	0.093					
Taste of pork		25.511	4	6.378	8.506	<0.001
Not at all good	-0.582					
Rather not good	0.001					
Neither/nor	-0.180					
Rather good	0.183					
Very good	0.578					
Safety of pork		4.793	4	1.198	1.598	0.173
Not at all safe	0.022					
Rather not safe	-0.232					
Neither/nor	-0.026					
Rather safe	0.038					
Very safe	0.198					
Healthiness of pork		13.879	4	3.470	4.628	0.001
Not at all healthy	-0.430					
Rather not healthy	-0.343					
Neither/nor	-0.085					
Rather healthy	-0.176					
Very healthy	1.034					

Multiple $R^2 = 0.238$

* Regression coefficient in the ANCOVA model for BMI

Table S3 Estimates of effects of predicting factors for beef consumption (General Linear Model with ANCOVA)

Factor	B	Type III SS	df	Mean squares	F-Ratio	*p*-Value
Constant	0.523					
Language region		5.323	2	2.662	5.240	0.006
German	0.073					
French	0.107					
Italian	-0.180					
Gender		3.054	1	3.054	6.012	0.015
Male	0.081					
Female	-0.081					
Household size		8.480	2	4.240	8.347	<0.001
1-person	-0.194					
2-person	0.015					
≥ 3-person	0.179					
BMI	0.019*	2.644	1	2.644	5.205	0.023
Taste of beef		8.011	4	2.003	3.943	0.004
Not at all good	1.383					
Rather not good	-0.325					
Neither/nor	-0.631					
Rather good	-0.262					
Very good	-0.165					
Safety of beef		3.171	4	0.793	1.560	0.184
Not at all safe	-0.352					
Rather not safe	-0.084					
Neither/nor	0.144					
Rather safe	0.086					
Very safe	0.206					
Healthiness of beef		7.563	4	1.891	3.722	0.005
Not at all healthy	-0.376					
Rather not healthy	-0.187					
Neither/nor	0.002					
Rather healthy	0.204					
Very healthy	0.357					

Multiple $R^2 = 0.158$

* Regression coefficient in the ANCOVA model for BMI

Table S4 Estimates of effects of predicting factors for poultry consumption (General Linear Model with ANCOVA)

Factor	B	Type III SS	df	Mean squares	F-Ratio	*p*-Value
Constant	0.496					
Language region		4.810	2	2.405	7.001	0.001
German	-0.037					
French	0.151					
Italian	-0.114					
Age	-0.007*	1.704	1	1.704	4.959	0.026
BMI	0.023*	4.574	1	4.574	13.314	<0.001
Taste of poultry		12.980	4	3.245	9.446	<0.001
Not at all good	-0.504					
Rather not good	-0.276					
Neither/nor	0.060					
Rather good	0.292					
Very good	0.428					
Safety of poultry		4.127	4	1.032	3.003	0.018
Not at all safe	-0.265					
Rather not safe	0.086					
Neither/nor	0.134					
Rather safe	0.178					
Very safe	-0.133					
Fat content of poultry		1.996	4	0.499	1.452	0.215
Not at all fat	0.036					
Rather not fat	0.009					
Neither/nor	-0.089					
Rather fat	-0.149					
Very fat	0.193					
Preparation effort		2.833	4	0.708	2.062	0.085
Not at all easy	0.147					
Rather not easy	-0.029					
Neither/nor	-0.133					
Rather easy	-0.062					
Very easy	0.077					

Multiple R^2 = 0.174

* Regression coefficient in the ANCOVA model for BMI and Age
